# Novel clip method for endoscopic submucosal dissection defect closure reducing submucosal dead space in antithrombotic gastric patients

**DOI:** 10.1055/a-2223-4475

**Published:** 2024-01-17

**Authors:** Taisuke Inada, Yorinobu Sumida, Hitoshi Homma, Kosuke Maehara, Kazuo Shiotsuki, Shin-ichiro Fukuda, Hirotada Akiho

**Affiliations:** 1 37060 Department of Gastroenterology, Kitakyushu Municipal Medical Center, Kitakyushu, Japan

Wound suture after endoscopic submucosal dissection (ESD) is anticipated to be beneficial in the stomach, particularly for patients taking antithrombotic medications, as they face an increased risk of posterior bleeding 1 2 . However, defect closure in the stomach presents challenges due to its relatively thick mucosa and muscular layer 3 . This was achieved without leaving any submucosal dead space by using a novel clip that features sharp claws at the tip for a strong grasping force ( Fig. 1 ). The video shows the procedure ( Video 1 ).

**Fig. 1:**
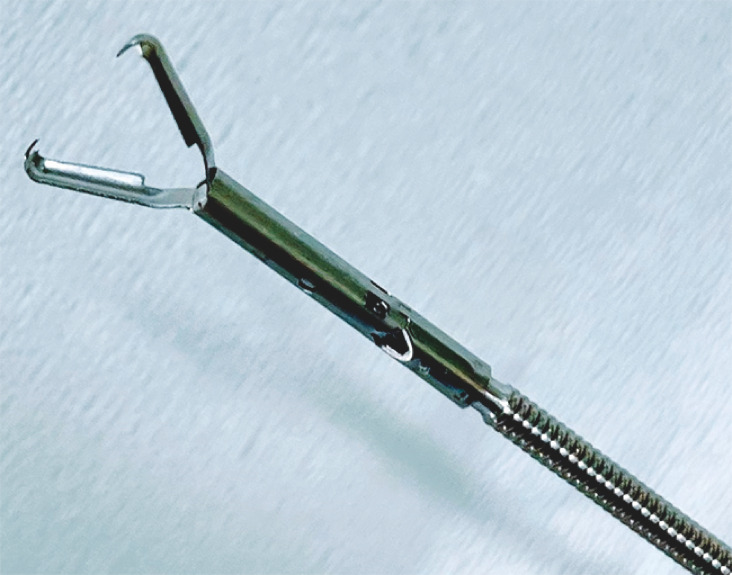
A novel clip with sharp anchor that can be rotated and regripped.

Suturing technique without submucosal dead space: a new clip for treating post-endoscopic submucosal dissection wounds on the anterior wall of the gastric antrum in patients taking antithrombotic drugs.Video 1

A man in his 80s with a history of stroke taking 30 mg/day of edoxaban underwent ESD for a 20-mm lesion on the anterior wall of gastric antrum. Edoxaban was discontinued only on the day of the treatment. Before suturing, the visible vessels were cauterized using hemostatic forceps. The clip was used to grasp the muscle layer in the middle of the wound margin and then brought to the contralateral side of the margin ( Fig. 2 **a** ). Once close enough, the muscle layer was pulled in with suction and then clipped ( Fig. 2 **b,c** ). The top and bottom of the first clip were clipped in the same manner ( Fig. 2 **d,e** ). The wound was sutured to ensure that the muscle layer was crimped, preventing any occurrence of submucosal dead space. Additional regular clips were used to close the space between the previously placed clips ( Fig. 2 **f** ). Following ESD, the patient resumed taking edoxaban on the day after the procedure without experiencing any posterior bleeding or delayed perforation.

**Fig. 2:**
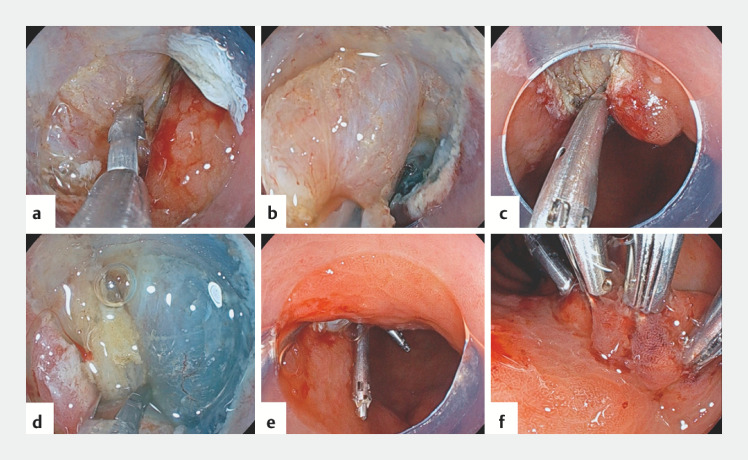
**a–c** The clip was used to grasp the muscle layer in the middle of the wound margin and then brought to the contralateral side of the margin. Once close enough, the muscle layer was pulled in with suction and then clipped. **d,e** The top and bottom of the first clip were clipped in the same manner. **f** Additional regular clips were used to close the space between the previously placed clips.

While there have been several reports of suture methods using clips or endoscopic instruments, these approaches tend to cause submucosal dead space because they draw the mucosal layer 4 5 . The method minimizes submucosal dead space by crimping the muscular layer instead of pulling the mucosa. This technique is valuable for achieving strong wound closure in the stomach.

Endoscopy_UCTN_Code_TTT_1AO_2AC

## Notes

Endoscopy E-Videos https://eref.thieme.de/e-videos
*E-Videos* is an open access online section of the journal *Endoscopy* , reporting on interesting cases and new techniques in gastroenterological endoscopy. All papers include a high-quality video and are published with a Creative Commons CC-BY license. Endoscopy E-Videos qualify for HINARI discounts and waivers and eligibility is automatically checked during the submission process. We grant 100% waivers to articles whose corresponding authors are based in Group A countries and 50% waivers to those who are based in Group B countries as classified by Research4Life (see: https://www.research4life.org/access/eligibility/ ). This section has its own submission website at https://mc.manuscriptcentral.com/e-videos .
